# The pathogenic effects of particulate matter on neurodegeneration: a review

**DOI:** 10.1186/s12929-022-00799-x

**Published:** 2022-02-22

**Authors:** Ran You, Yuen-Shan Ho, Raymond Chuen-Chung Chang

**Affiliations:** 1 Nanjing Key Laboratory of Pediatrics, Children’s Hospital of Nanjing Medical University, 72 Guangzhou Road, Nanjing, 210008 China; 2 School of Nursing, Faculty of Social and Health Sciences, The Hong Kong Polytechnic University, Hung Hom, Kowloon, Hong Kong SAR China; 3 Laboratory of Neurodegenerative Diseases, School of Biomedical Sciences, LKS Faculty of Medicine, Pokfulam, Hong Kong SAR China; 4 State Key Laboratory of Brain and Cognitive Sciences, The University of Hong Kong, Pokfulam, Hong Kong SAR China

**Keywords:** Particulate matter, Neurodegeneration, Alzheimer’s disease, Cognitive dysfunctions, Oxidative stress, Neuroinflammation

## Abstract

The increasing amount of particulate matter (PM) in the ambient air is a pressing public health issue globally. Epidemiological studies involving data from millions of patients or volunteers have associated PM with increased risk of dementia and Alzheimer’s disease in the elderly and cognitive dysfunction and neurodegenerative pathology across all age groups, suggesting that PM may be a risk factor for neurodegenerative diseases. Neurodegenerative diseases affect an increasing population in this aging society, putting a heavy burden on economics and family. Therefore, understanding the mechanism by which PM contributes to neurodegeneration is essential to develop effective interventions. Evidence in human and animal studies suggested that PM induced neurodenegerative-like pathology including neurotoxicity, neuroinflammation, oxidative stress, and damage in blood–brain barrier and neurovascular units, which may contribute to the increased risk of neurodegeneration. Interestingly, antagonizing oxidative stress alleviated the neurotoxicity of PM, which may underlie the essential role of oxidative stress in PM’s potential effect in neurodegeneration. This review summarized up-to-date epidemiological and experimental studies on the pathogenic role of PM in neurodegenerative diseases and discussed the possible underlying mechanisms.

## Background

Inhalable particulate matter (PM) is a mixture of particles suspended in the air, which may be directly released into the air as combusted diesel/gasoline from vehicles, mineral dust, industrial emissions, or generated through chemical reactions of other airborne pollutants, such as nitrogen oxides, heavy metals, and organic compounds [1, 2]. Apart from occupational exposure among construction or printer workers and exposure in disasters and war, the most common sites for PM exposure are roads with heavy traffic [3]. PM’s size, composition, surface properties, and concentration vary with its source. One of the most well-accepted classifications of PM is based on the aerodynamic diameter and divides PM into PM10 (median particle diameter ≤ 10 μm), PM2.5 (median particle diameter ≤ 2.5 μm) and PM0.1 or ultrafine particulate matter (UFPM, median particle diameter 0.1 μm) [1]. Among these, PM2.5 and PM0.1 are predominantly harmful because of their ability to penetrate tissues deeply and the difficulty involved in their clearance [1, 2, 4, 5].

The harmful effects associated with PM have become an increasingly severe global issue. Billions of people around the world are chronically exposed to air pollution above the promulgated safety standards [2]. Recently, the Independent Particulate Matter Review Panel of the Environmental Protection Agency Clean Air Scientific Advisory Committee recommend a strict standard for PM control on *the New England Journal of Medicine* because lowering the limit of PM2.5 by 3 μg/m^3^ (12 to 9 μg/m^3^) was estimated to reduce the risk of all-cause mortality from short-term exposure to PM2.5 by up to 27% [6]. Apart from limiting PM2.5 emissions, preventing or treating the adverse health outcomes caused by PM2.5 exposure is also a critical issue that remains to be addressed.

Numerous studies have associated the inhalation of PM with increased onset and mortality of diseases involving the pulmonary [7, 8], cardiovascular [9], immune, and central nervous systems (CNS) [10, 11]. These adverse effects on health are due to the broad distribution of PM2.5 and UFPM in the body. In recent decades, evidence from numerous epidemiological and experimental studies have indicated PM as a risk factor for neurodegenerative diseases, especially Alzheimer’s disease (AD) and Parkinson’s disease (PD) [10, 11]. AD is characterized by progressive cognitive impairment, accompanied by neuronal death, neuroinflammation, and the accumulation of two neuropathological markers, i.e., senile plaque composed of amyloid-β (Aβ) peptides and neurofibrillary tangles accumulated from hyperphosphorylated tau [12]. Patients with PD symptomatically manifest motor deficits due to the loss of dopaminergic neurons. PD is pathologically characterized by Lewy bodies formed by α-synuclein aggregation [13]. It is estimated that 40% of the adults older than 85 years old have AD and 10% have PD, placing a great economic burden on our aging society [13, 14]. Determining the risk factors for AD is crucial because most of the patients have sporadic AD, and there lacks effective treatments to cure or slow down its progression [14]. Therefore, identifying the environmental risk factors may help develop better preventive measures for AD and PD.

In this review, we analyzed what we know so far about the onset and prognosis of neurodegenerative diseases, neurodegenerative-like behavioral and pathological changes associated with PM in human studies and animal studies. Firstly, we provided the evidence of the existence of PM in human brain and introduced the modes of their entry to the brain. Secondly, we summarized the studies on the association of PM with the onset, pathology and prognosis of neurodegenerative diseases and PM’s adverse effect on cognition and neurodegenerative-like pathology. Thirdly, we summarized the effects of PM on neurons, synapses, neuroinflammation, the blood–brain barrier (BBB), and neurovascular units (NVU), which are key pathological features of neurodegeneration that contribute to the disease’s progression. Finally, we described the subcellular effects of PM on organelles including the mitochondria and endoplasmic reticulum (ER).

## Evidence of PM’s existence in the brain

The deposition of PM in the brain is an emerging issue. Nanosized PM has been found in human olfactory bulb periglomerular neurons, and particles < 100 nm have been observed in intraluminal erythrocytes of the frontal lobe and trigeminal ganglia capillaries in the human brain [15]. More direct evidence of PM’s existence in the brain was recently obtained using magnetometry, high-resolution transmission electron microscopy (HRTEM), electron energy loss spectroscopy, and energy-dispersive X-ray to analyze the nanoparticles deposited in the frontal cortex of residents in Metropolitan Mexico City (MMC) and Manchester [16]. This study was first reported about rounded nanoparticles in human brains. They analyzed these nanoparticles’ mineralogy, morphology and composition. The study concluded that these nanoparticles in the human brain were infiltrated from an external source such as PM as they showed distinct properties in surface textures and size distribution comparing to endogenously formed and biogenic nanoparticles. Besides, chemical composition analysis revealed that these brain magnetites were similar to the airborne PM generated by combustion of fossil oil [16]. However, the authors did not quantify the amount of PM that deposited in the brain, nor confirmed the exposure route of PM.

## Modes of PM’s entry into the brain

Nanoscaled PM can enter the brain through the olfactory system, the BBB from the systemic circulation, or in a less extent, the trigeminal or facial nerves [17].

### The olfactory system route

The olfactory system provides a nose-to-brain route for inhaled particles. In the olfactory epithelium the primary olfactory neurons contact with the environment and project to the olfactory bulb via axons [18]. Foreign substances, such as airborne nanosized inorganic particles, may enter the brain via olfactory nerves in a retrograde manner [19]. This pathway has been proved by using autoradiography and gamma spectrometry in the olfactory bulb of rats that were exposed to manganese (_54_Mn^2+^) via intranasal instillation. In this study, the metal was initially uptake by the olfactory bulb and then migrate via secondary and tertiary olfactory pathways and via further connections into most parts of the brain and also to the spinal cord [18]. Recently, the transportation of aerosol UO_4_ in rats was also studied by using inductively coupled plasma mass spectrometry to detect the concentration of UO_4_ in brain regions and using secondary ion mass spectrometry microscopy and transmission electron microscopy coupled with energy-dispersive X-ray spectroscopy to track elemental uranium in situ in the olfactory epithelium. Uranium deposited in the brain regions including olfactory bulbs, frontal cortex, hippocampus, cerebellum, and brainstem in the exposed rats. More precisely, elemental uranium was detected in anatomical regions including olfactory neuron dendrites, paracellular junctions of neuroepithelial cells, and olfactory nerve tracts (around axons and endoneural spaces) [20]. By utilizing the nose-to-brain transportation pathway, chitosan-based nanoparticles, polyester-based nanoparticles, solid lipid nanoparticles, liposomes, nanoemulsions, micelles, and nanocomplexes were applied for brain delivery of intranasally administrated drugs [21].

Although the deposition of airborne ultrafine nanoparticles in the olfactory system was limited to < 3.5% in humans [22], the olfactory bulb seems to be primarily affected by airborne PM with a size of approximately 100 nm [23]. The levels of the neurotransmitters glutamate and glycine, and proinflammatory cytokines increased in the olfactory bulb of mice 6 and 11 h after a single inhalation of nanosized carbon black, respectively [24]. Excessive excitatory neurotransmitters and proinflammatory cytokines may be neurotoxic and pro-inflammatory. These findings demonstrated that the olfactory bulb was rapidly affected by nanosized carbon black, a component of PM, indicating that the olfactory system was more than a route for the nanoparticles and PM to enter the brain, the impairment in the olfactory bulb may also be part of the neurodegenerative pathology. Consistent with the findings in the experimental study, a study on the young adult citizens in MMC found association between air pollution exposure and olfactory dysfunction and pathology in the olfactory bulb [25]. Meanwhile, the young adults living in MMC demonstrated olfactory bulb endothelial hyperplasia, neuronal accumulation of particles, and immunoreactivity to Aβ [42] and/or α-synuclein in neurons, glial cells and/or blood vessels. There were ultrafine particles deposited in the endothelial cytoplasm and basement membranes of the olfactory bulb in the young adult citizens in MMC.

### The trigeminal nerve route

The nasal cavity is innervated by the maxillary division of the trigeminal nerve that projects to the spinal trigeminal nucleus. The transportation route of aerosol particles via trigeminal nerve was studied in rats and mice that were exposed to MnCl_2_ aerosols. By using proton induced X-ray emission to study the concentration of Mn in tissue, there were statistically elevated levels of Mn in trigeminal ganglia 0-, 7- and 14-days after nose-only exposure and a small but significant increase of Mn in the spinal trigeminal nucleus 7-days after exposure [26]. This finding indicated that the trigeminal nerve could serve as a pathway for entry of inhaled Mn to the brain in rodents following nose-only exposure.

### The circulation and BBB route

A single inhalation of ultrafine ^13^C particles (^13^C UFP), a mimic of UFPM, for 6 h resulted in a significant and persistent increase in ^13^C in the olfactory bulb, and a significant but fluctuated increase in the cerebrum and cerebellum of rats until 7 days after the exposure. This finding indicated that UFPM and nanoparticles may have two ways to enter the brain, i.e., a direct access through the olfactory route and an indirect access through circulation [23]. The inhaled nanosized particles can enter the circulation after penetrating deep into the alveolar region of the lungs as free particles, or they may enter the lymph after being engulfed by the phagocytotic cells such as the pulmonary macrophages [17]. Ultrafine particles rapidly enter the circulation as demonstrated in a study that exposed human to ultrafine 99mTechnetium-labeled carbon particles via respiration [27]. By measuring the radioactivity of the blood sample after the inhalation of these particles, the ultrafine particles entered the circulation only 1 min after inhalation [27].

After systemic absorption, the nanoparticles could enter the brain by penetrating the BBB [19, 28, 29]. The BBB is comprised of tightly bound endothelial cells and regulates the material transportation between the circulation and brain. The indoor PM2.5 effectively crossed a 3D human BBB organotypic chip, which was a co-culture of human endothelial cells and astrocytes in microfluidic chamber. They also found cytotoxicity and an abnormal proliferation in astrocytes post PM2.5 exposure in the chip, which was likely attribute to oxidative stress [30]. After penetrating the BBB, PM impairs the BBB’s structural integrity and increases its permeability by inducing cytotoxicity in the endothelial cells and neuroinflammation [31], as seen in the brains of children in MMC [32, 33].

## PM was associated with increased risk of dementia

### Human studies

Recent epidemiological studies reported that PM exposure was correlated with an increased risk of the onset of dementia [34, 35] and AD [34], the most common neurodegenerative diseases that is featured with cognitive impairment (Table 1). For instance, the incidence of dementia caused by AD or unspecified cause in Europe were correlated with air pollution especially PM2.5 and NO [36]. The estimated incidence rate (cases per year) of AD and dementia with unspecified cause that were associated with air pollution exposure over Europe was 498,000 and 314,000, respectively [36]. The situation was expected to be continuously worse because there was an around 72% increase in the future incidence rate for both types of dementia when considering the effect of climate change and the foreseen population aging in Europe [36]. In the United States, exposure to PM2.5 contribute to the racial disparities in the AD risk in women according to a follow-up study with 158 incident cases (21 in Black women) [37]. The estimated PM2.5 exposure was slightly higher in Black than in White women, and the observed association between PM2.5 and AD risk was ~ 2 times greater in Black than in White women [37]. Moreover, high PM2.5 level was associated with significantly increased risk of AD mortality and hospital admission in seniors (aged 65 +) [38]. These findings from data obtained from millions of patients suggested that high PM2.5 exposure level was associated with increased risk of dementia/AD onset and poorer prognosis.

**Table 1:** Summary of the epidemiological studies on the association between PM/airborne pollution and onset/prognosis of dementia

Type of PM	Cohort size	Gender	Area	Age	Tests	Dysfunction that correlated with PM exposure	References
PM2.5	1,807,133	Male (44.91%); Female (55.09%)	Canada	65 years and older	A validated algorithm combines relevant physician claims, hospital admissions and prescription drug use	Onset of dementia (hazard ratio 1.016)	[35]
PM2.5 (from residential wood burning/ vehicle exhaust)	1806	Male (773); Female (1033)	Sweden	55–85 years	Health questionnaire and cognitive evaluation	Onset of dementia (hazard ratio 1.55)	[34]
PM2.5	-	Male and female	Europe	55–80 + years old	Exposure–response functions	The incidence of dementia	[36]
PM2.5	6485	Female	America	65–79 years	WHIMS; the Modified MMSE; cranial computerized axial tomography scan and laboratory blood tests	Racial/ethnic disparities in AD risk	[37]
PM2.5	18,178	Male 8845 (48.7%); Female 9333 (51.3%)	America	75.8 ± 6.3 years	Amyloid PET scan, MMSE	Aβ plaques	[61]
PM2.5	2,022,647 person-years	Study group: Female (58.9%); Control group: Female (55.0%)	America	65 + years	Health data obtained from the State Center for Health Statistics and the Healthcare Cost and Utilization Project	Mortality of AD	[38]
PM2.5	83,343	Cases: Male (72%); Female (28%) Controls: Male (60%); Female (40%)	America	12–92 years	Follow-up interviews	Parkinson's disease risk	[72]

PET: positron emission tomography; MMSE: Mini-mental State Examination; WHIMS: Women´s Health Initiative Memory Study; AD: Alzheimer’s disease

Abundant evidence from epidemiological studies have demonstrated that PM, especially PM2.5, is correlated with the decline of cognitive performance in people of various age groups who are chronically exposed to high levels of PM2.5 (Summarized in Table 2).Chronic exposure to PM2.5 impaired motor development in infants [39] and retarded the development of working memory in school children in Spain [40]. However, in a prospective birth cohort study in eastern Massachusetts (the United States) which included 1109 mother–child pairs, PM2.5 exposure was not associated with poorer cognitive performance [41]. The impact of PM2.5 in cognitive development may have sex difference in children. In a population-based birth cohort study in Spain (n = 1119), PM was negatively correlated with memory in boys; however, it showed no significant correlation with cognition without stratifying the gender [42]. Although these cognitive developmental retards in children may not be a symptom of neurodegeneration, environmental pollutant exposed in early life may not only induce instant brain function disorder but also increase the susceptibility to late-onset diseases such as neurodegeneration whose onset and rapid progression are strongly correlated with environmental risk factors [10, 43]. Indeed, the correlation between PM exposure and the cognitive decline in the adults, especially in those older than 50 years of age, was prominent as revealed by a series of studies including thousands of participants from the United States [44–48], Europe [49] and China [50]. These cognitive impairments included working memory deficits [46] and episodic memory loss, the hallmark symptoms of preclinical AD [47], and mild cognitive impairment (MCI), which is the intermediate state between normal cognitive aging and dementia [49].

**Table 2:** Summary of the epidemiological studies on the association between PM and cognitive decline

Type of PM	Cohort size	Gender	Area	Age	Tests	Cognitive function tested	Cognitive dysfunction that correlated with PM exposure	References
PM2.5	438	Male 198 (45.2%); Female 240 (54.8%)	Spain	Around 15 months (range 13–18 months)	Bayley scales of infant development	Mental scores and motor scores	Motor development impairment	[39]
PM2.5	1439	Male 743 (51.6%); Female 696 (48.4%)	Spain	11.4 ± 0.6 years (age at the last follow-up)	N-back test	Working memory	Wording memory developmental impairment	[40]
PM2.5	1109	Male 554 (50%); Female 555 (50%)	America	8.0 years	Kaufman Brief Intelligence Test; Wide Range Assessment of Visual Motor Abilities; Wide Range Assessment of Memory and Learning	Verbal IQ, Nonverbal IQ, Visual motor, Design memory, Picture memory	None	[41]
PM2.5	1119	Male (50%); Female (50%)	Spain	4–6 years	McCarthy Scales of Children's Abilities	Verbal, Perceptive-Manipulative, Numeric, General cognitive, Memory and Motor	Memory and verbal impairment in boys, not in girls	[42]
PM2.5	780	Male (39%)、Female (61%)	America	55–64 years: (49%) 65–74 years: (25%) 75–84 years: (19%) 85 + years: (7%)	the Short Portable Mental Status Questionnaire	Cognitive function was assessed with a serial 3’s subtraction test to measure working memory and recall of the date, day of the week, and name of the president and vice-president to measure orientation	Impairment	[46]
PM2.5	13,996	Male (43.92%)、Female (56.08%)	America	64.0 ± 10.4 years (range 50–102 years)	Health and Retirement Study	Episodic memory score; Mental status score	Impairment	[44]
PM2.5	1496	Male 308 (20.6); Female 1188 (79.4%)	America	60.5 ± 8.1 years	California Verbal Learning Test, immediate recall and delayed recall	Verbal learning	Impairment	[45]
PM2.5 and PM10	5330	Male 1751 (32.9%); Female 3579 (67.1%)	America	75.2 ± 6.46 years	Neuropsychological test: Selective Reminding Test; (Color Trails 2-Color Trails 1), Controlled Oral World Association Test; Identities and Oddities; similarities subtest from the Wechsler Adult Intelligence Scale; Boston Naming Test [15-item], Animal Naming	Global cognition score; memory; executive function; language	Impairment	[48]
PM2.5	998	Female	America	73–87 years	California Verbal Learning Test	Episodic memory	Impairment	[47]
PM2.5	2050	Male 1007 (49.1%),Female 1043 (50.9%)	Germany	64.1 ± 7.7 years	Cognitive performance assessment	Immediate and delayed verbal memory, problem solving/speed of processing, verbal fluency and abstraction/visual–spatial organization	Impairment	[49]
PM2.5	13,324	Male 6,334 (47.5%); Female 6,990 (52.5%)	China	82.4 ± 11.9 years	MMSE	Orientation, registration, attention, memory, language, and visual construction skills	Impairment	[50]
PM2.5	2253	Female:1430 (63.5%)	Sweden	72.1 ± 9.9 years	MMSE	The speed of cognitive decline	accelerated cognitive decline	[112]
PM2.5 and PM10	7311	Male: 3680 (51.3); Female: 3631 (49.7)	China (rural area)	68.6 ± 6.9 years	Chinese version of MMSE	Orientation function, memory, attention/concentration function, language function, and visuospatial function	Impairment	[113]

*MMSE* Mini-mental State Examination

### Animal studies

In an animal study, it was found that exposure to ambient air pollution both pre- and postnatally induced short-term memory deficit as assessed by the novel object recognition test in offspring, indicating that the exposure to PM2.5-enriched air leads to cognitive impairment [51]. Young adult mice that inhaled PM2.5 for 10 months showed impairment in spatial learning and memory [52]. UFPM exposure for 2 weeks at human-relevant concentration resulted in memory deficits in reference memory and short-term memory since 1 month to 6.5 months post-exposure in both nontransgenic mice and aged male 3 × TgAD mice, a transgenic mice model for AD that manifested deposition of AD pathological hallmarks and cognitive impairment [53]. Although in another study which continuously exposed 6-month-old female 3 × TgAD mice to real world PM2.5 for 3 months, the mice did not show cognitive decline in Morris water maze test, PM2.5 induced obvious neuronal loss accompanied with phosphorylated tau in the olfactory bulb and the hippocampus in the cortex of exposed mice [54]. (Table 3).

**Table 3:** Summary of the animal studies on the association between PM and cognitive decline

Type of PM	Exposure duration	Species	Gender	Genotype	Age	Tests	Cognitive function	Cognitive impaired or not	References
Cognition									
PM2.5	150 days	Wistar rats	Male	Wild type	45 days old	The spontaneous nonmatching-to-sample recognition test	Discriminative memory and habituation	Impaired	[51]
PM2.5	10 months	C57BL/6 mice	Male	Wild type	4-week-old	Barnes maze	Learning and memory	Impaired	[52]
UFPM	2 weeks	Mice	Male	3 × TgAD mice	12.5- month-old	Radial arm maze; Novel object recognition test	Spatial memory; Short-term memory	Impaired	[53]
PM2.5	3, 6, 12-months Intratracheally injection of 20 mg/kg PM2.5 every 7 days	Sprague–Dawley rats	Male	Wildtype	2-month-old	Morris water maze test; Tail flick and hot plate test	Spatial learning and memory; Sensory function	Impaired	[103]
PM2.5	3 months	Mice	Female	3 × TgAD mice	6- month-old	Morris water maze	Learning and memory	Not impaired	[54]

Type of PM	Exposure duration	Species	Gender	Genotype	Age	Abnormality	Potential regulating signals	Treatment	References
Neurodegenerative-like pathology									
Artificial PM	4.5 months	Wistar rats	–	Wild type	~ 5 weeks of age	Neuronal loss: pure cortical neuronal loss, selective neuronal loss, nuclear pyknosis, karyolysis, and karyorrhexis	–	–	[59]
UFPM	8 days	C57BL6/J mice	Male and female	Wild type	8-week-old	Ventriculomegaly	–	–	[60]
UFPM	2 weeks	B6/129 hybrid mice	Male	3 × Tg AD	12.5–14 months	Aβ deposition and neuroinflammation	–	–	[62]
PM2.5	9 months	C57BL/6 mice	Male	Wild type	8-week-old	Increased Aβ1-40	BACE/APP/Aβ	–	[63]
PM2.5	4 weeks	C57BL/6 mice	Male	Wild type	8-week-old	Cognitive impairment Synaptic dysfunction Neuroinflammation	NF-κB/miR-574-5p/BACE1	miR-574-5p or BACE1 knockdown	[64]
Oxidative stress									
UFPM/PM2.5	3 weeks/60 min	C57BL6 mice/Wistar rats	Male	Wild type	11–12 months old/90 days old	Oxidative stress in hippocampus	redox homeostasis	–	artificial UFPM (no oxidant): [74]; PM2.5: [75]
UFPM	8 weeks	Sprague–Dawley rats	Male	Wild type	6-week-old	Oxidative stress, neuroinflammation, and ER stress in the striatum	Nrf2/HO-1 signaling; NF-κB signaling; XBP-1/Bip	–	[76]
PM2.5	28 days	C57BL/6 mice	Male	*Nrf2*^−/−^	–	Severe neuronal injury in the olfactory bulb	Nrf2	Nrf2-mediated defenses against oxidative stress	[78]
Neuroinflammation									
Concentrated ambient PM	6 weeks	C57BL/6J mice	Male	*ApoE*^*−/−*^	6-week-old	Neuroinflammation	MAP kinase signaling pathways	–	[86]
Nanosized PM (diameter < 0.2 μm)	10 weeks	C57BL/6J mice	Female	Wild type	3-month-old	Brain inflammatory responses	TLR4	–	[88]
Nanosized PM (diameter < 0.2 μm)	10 weeks	C57BL/6J mice	Male	Wild type	15–16 weeks	Increased complement C5/C5α protein and CD88; Activated microglia in the corpus callosum	C5/C5α complement pathway	–	[91]
BBB & NVU injury									
Mixed vehicle exhaust	30 days	C57BL/6 mice	Male	*ApoE*^*−/−*^	12-week-old	BBB permeability increase; Decreased tight junction proteins	–	–	[95]
PM and ozone mixture	4 h	Fischer-344 rats	Male	Wild type	–	Dysregulation of vasoregulatory pathways (ET-1 & iNOS mRNA expression)	–	–	[100]

### PM exposure may be directly associated with cognitive impairment

Since PM exposure could induce tissue injury and potential adverse effects on peripheral organs. Whether PM’s adverse impact on behavioral and CNS function was directly caused by PM or an indirect consequence that comes after peripheral organ injury was an issue that worth investigation and discussion.

In a prospective epidemiological study that used longitudinal observational data for 49,705 people aged 18 + from 2006 to 2015 from the Dutch Lifelines cohort study, the researchers tested the direct/indirect associations between PM exposure and cognitive decline by using linear structural equation modeling [55]. They found that higher exposure to PM was related to worse cognitive function, and the direct association of PM constituted more than 97% of the total effect. This study suggested that PM exposure was mainly directly associated with cognitive decline.

Some animal studies provided experimental data for the question of whether the effect of PM on peripheral organs affected the conclusion of PM’s influence on the CNS function. For instance, chronic PM inhalation may impair the cardiopulmonary function, which may affect the animal’s performance in some cognitive behavioral tests, such as Barnes maze. Fonken et al., tested the physical measurements and cognition of the mice that were chronically exposed to PM2.5 for 10 months [52]. They found that long-term exposure to PM2.5 impaired the cognition without affecting the mice’s body mass, body length, vibrissae, eye appearance, gross olfactory abilities, muscle tone, sensorimotor responses, motor performance and serum corticosterone concentration [52]. These findings in human and animal studies hence suggested that PM2.5 exposure may impaire cognition in a direct manner.

## PM may induce neurodegenerative pathology

Along with cognitive decline, neuropathological changes were also found in the brains of adults living in heavily polluted urban areas. In a study having 10,343 9- and 10- year-old children living in the US, the annual PM exposure concentration was associated with hemispheric specific differences in gray matter across cortical regions, subcortical regions, and cerebellum [56]. Older women with increased exposure to PM2.5 had significantly smaller white matter volume in the frontal lobe, temporal lobe, and corpus callosum [57]. They also showed global gray matter atrophy [47]. In a longitudidal study on the brain MRI of 236 participants from 2004 to 2010, low PM2.5 (annual average level below the Environmental Protection Agency standard) was not associated with MRI indicators for small vessel disease or neurodegeneration, including brain parenchymal fraction, white matter hyperintensities, and cerebral microbleeds [58], indicating limiting PM2.5 level beneath the standard may not contribute to the pathology of neurodegeneration. Artificial PM dose-dependently induced different patterns of neuronal loss, including pure cortical neuronal loss, selective neuronal loss, nuclear pyknosis, karyolysis, and karyorrhexis in rats [59]. In mice, UFPM exposure early in life induced permanent ventriculomegaly, that is, enlargement of the lateral ventricles, which is also seen in autism and schizophrenia [60].

Higher PM2.5 concentrations were associated with brain Aβ plaques according to a cross-section study which analyzed the amyloid positron emission tomography scan data of 18,178 patients with MCI or dementia [61]. Similarly, PM exposure exacerbated AD-like neuropathology, including Aβ deposition and neuroinflammation in the brains of young adults and children living in Mexico City Metropolitan Area (MCMA), an urban area heavily polluted with PM2.5 [15], and in UFPM-exposed aged 3 × Tg AD mice, an transgenic mouse model that mimicked the AD neuropathology [62]. Aβ was produced by the cleavage of amyloid precursor protein (APP) by its proteolytic enzyme, beta-site amyloid precursor protein cleaving enzyme (BACE). Nine months-exposure to PM2.5 increased Aβ1-40 and BACE, and decreased APP in mice brains [63]. BACE1 may contribute to the neuroinflammation, synaptic dysfunction, and cognitive impairment induced by 4-week exposure to PM2.5 in C57BL/6 mice [64]. BACE1 may be upregulated when miR-574-5p, whose target gene is BACE1 was downregulated by nuclear factor-κB (NF-κB) p65 [64]. Restoring miR-574-5p level in the hippocampus or knockdown BACE1 could effectively decrease BACE1, protect the synapse, and improve cognition, learning, and memory post exposure to PM2.5 [64]. This study emphasized the pathogenic role of BACE1 in the synaptic toxicity and cognitive decline induced by exposure to PM2.5 and demonstrated a potential regulation via NF-κB/miR-574-5p signaling [64].

## PM may induce neurotoxicity, oxidative stress, neuroinflammation, and impairs BBB: potential pathogenic effects of PM on neurodegeneration and cognitive impairment

### Neurotoxicity

Exposure to PM induces prominent cognitive decline in humans and laboratory animals due to structural changes and atrophy in the gray and white matter [47, 57]. The hippocampus and glutamatergic neurons played crucial roles in the learning and memory; while PM exposure affected these neurons structurally and functionally. Glutamatergic neurons are susceptible to the insults of PM because oxidative stress induced by PM aggravates the glutamatergic excitotoxicity in neurons [65]. PM2.5 dose-dependently decreased the cell viability of primary human neurons and increased the levels of cleaved caspase-3 [66]. A 48-h treatment with nano scaled PM inhibited the neurite outgrowth in a primary culture of rat hippocampal neurons [67], and increased the neurotoxicity of NMDA (N-methyl-d-aspartic acid), a glutamatergic agonist, in hippocampal slice cultures [67]. These toxicities were blocked by AP5 [67, 68], an NMDA receptor antagonist, indicating that PM-induced neurotoxicity was mediated by the NMDA receptor. Neurons in the cornu ammonis area 1 (CA1) of the hippocampus may be more sensitive to PM-induced neurotoxicity. Exposure to aqueous nanosized PM for 2 h altered the postsynaptic proteins in this region in a hippocampal slice culture, including increase in GluA1, NMDA receptor units such as GluN2A and GluN2B, and post-synaptic density protein 95 and spinophilin; while neurons in the dentate gyrus were unresponsive to the PM’s stimulation [68].

Functionally, exposure to PM2.5 increased the amplitude of field excitatory postsynaptic potentials in hippocampal brain slices, indicating enhanced excitatory synaptic transmission and excitatory neurotoxicity [65]. Nanosized PM decreased the evoked excitatory postsynaptic currents of CA1 neurons. These results demonstrated that the exposure to PM changed the structure and function of the hippocampal glutamatergic neurons, especially those in the CA1 region [67, 68]. An in vivo study showed contrary results, which showed a decrease in GluA1 in the total lysate of the hippocampus in young C57BL6/J mice exposed to nanosized PM for 10 weeks [67]. Further investigation may be required before achieving a conclusion.

Dopaminergic neurons are particularly vulnerable to oxidative stress. This is not only because they support a synaptic network with a high energy demand but also because the metabolism of dopamine itself generates ROS. PM size-dependently induced cell loss in N27 cultures, a rat dopaminergic neuron cell line, and in the mixed culture of the striatum. These PM induced-neuronal loss was accompanied by a significant increase in reactive nitrogen species, indicating PM may impair the dopaminergic neurons via oxidative stress [69]. Similarly, the PM-caused cytotoxicity in SH-SY5Y cells, a dopaminergic-like neuronal cell line, was accompanied by the generation of the superoxide anion (O_2_^−^) [70]. PM may indirectly damage dopaminergic neurons by activating microglia to release superoxides. Ultrafine carbon black could further aggravate the toxicity of rotenone in dopaminergic neurons in a mixed culture of neurons and glia from the midbrain by inducing superoxides in microglia after being recognized by the surface receptor MAC-1 [71]. Indeed, an epidemiological study showed that the concentration of PM2.5 was positively associated with the incidence of PD in North Carolina [72]. Hence, these results from in vitro studies and epidemiological studies suggest that exposure to PM may be a risk factor for PD.

These studies demonstrate that glutamatergic neurons in the hippocampal subfields, the integrity and function of which are critical for normal cognitive behavior, and dopaminergic neurons, the injury to which was the primary pathogenic factor leading to PD, were susceptible to the effects of PM. Further investigation of the neurotoxic mechanisms is essential to develop therapeutic interventions against the neurotoxicity caused by PM.

### Oxidative stress

Oxidative stress is one of the most common detrimental factors in the brain and plays a central role in the neurotoxicity caused by PM [10, 11]. PM induces oxidative stress when it contains oxidants [73]. A recent neurometabolomics analysis suggests that insoluble nanosized PM itself induced prominent oxidative stress because the mice brains used in the analysis were exposed to artificial UFPM, which did not contain toxic oxidants [74]. Among the brain regions, the hippocampus showed more profound metabolic disturbances than the olfactory bulb, cerebral cortex, or cerebellum [74]. Thus, disturbances in redox homeostasis in the hippocampus may be a primary pathogenic factor that leads to adverse effects by PM exposure in the brain. In an ex vivo study, homogenates of rat brain regions, including the olfactory bulb, cerebral cortex, striatum, hippocampus, and cerebellum, were incubated with a suspension containing PM2.5. Consistent with the findings from the neuro-metabolic study, the hippocampus and cerebellum are more vulnerable than other regions to the oxidative stress induced by PM2.5 [75]. These results highlighted a crucial role of oxidative stress in the hippocampus in the toxicity induced by PM in the brain.

Subsequently, oxidative stress induced glutamatergic excitotoxicity by upregulating cyclooxygenase 2 (COX-2) in the hippocampal neurons exposed to PM2.5 [65]. This finding associated oxidative stress with the excitatory neurotoxicity by PM in the hippocampal neurons. Relieving oxidative stress with reactive oxygen species (ROS) inhibitor N-acetyl-L-cysteine effectively suppressed the abnormally increased amplitude of field excitatory postsynaptic potentials in hippocampal brain slices incubated with PM2.5 [65], hence suggesting that ROS inhibitors may be a potential therapeutic strategy to treating the neurotoxicity of PM.

The nuclear factor (erythroid-derived2)-like2 (Nrf2) signaling pathway is the primary signaling pathway for the reversal of oxidative stress. Chronic exposure to UFPM induced the gene expression of detoxifying enzymes such as hemeoxygenase-1 through the activation of the Nrf2 signaling pathway in the olfactory bulb, frontal cortex, hippocampus, and striatum in rats [76] and in the cerebellum of young adult C57BL/6J mice [77]. These findings indicate that the activation of the Nrf2 pathway and its downstream detoxifying enzymes in the brain may be a stress response to PM. In *Nrf2*^−/−^ mice, severe neuronal injury was observed in the olfactory bulb after exposure to PM2.5 via intranasal instillation [78], indicating that the maintenance of the Nrf2-mediated redox homeostasis plays a vital role in relieving the neurotoxicity caused by the exposure to PM.

### Neuroinflammation

Neuroinflammation and oxidative stress are central mediators that form a vicious cycle leading to the adverse effects in the brain caused by exposure to PM [10, 11]. ROS, which is released by activated microglia, could trigger neuroinflammation as a pro-inflammatory mediator [79]. PM has robust pro-inflammatory effects not just because it contains lipopolysaccharides and other pro-inflammatory components [80] but also because the insoluble nanoparticles act as immune stimulants [29, 81, 82]. Increasing lines of evidence from epidemiological and experimental studies have found increased levels of cytokines, inducible nitric oxide synthase, and COX-2 in human brains [15], human cerebrospinal fluid [83], and experimental animal brains [15, 63, 84] accompanied with impaired cognitive function which is associated with the level of PM. The affected brain regions include the olfactory bulb [24], cortex [51, 85, 86], hippocampus [87], and striatum [76].

NF-κB and Toll-like receptor 4 (TLR4) are robustly activated in the PM affected brains as analyzed in a microarray analysis on nanosized mixed cortical glial cultures exposed to PM [88]. Consistently, NF-κB and TLR4 are activated in the homogenate of the cortex of apolipoprotein E knockout (*ApoE*^*−/−*^) mice exposed to UFPM for 6 weeks [86] and in the hippocampus of mice that were chronically exposed to PM [88], repectively. Knockdown of *Tlr4* gene in the glial culture attenuated inflammatory responses after exposure to PM [88]. Another study has suggested the potential implication of high-mobility group box 1 (HMGB1)-induced microglial activation and neuroinflammation in organic dust, which was a mixture of PM of varying sizes, microbes, and microbial products [89]. Organic dust induced the nuclear translocation of HMGB1 in the microglia, leading to its activation and the subsequent inflammatory responses [89]. Knockdown of HMGB1 or inhibiting its nuclear translocation could alleviate its activation even after exposure to organic dust [89]. While all the discussed studies showed the activation of microglia and their subsequent inflammatory responses in the PM-exposed brains, it is not clear whether PM deposited in the brain displayed direct stimulation, or PM in the body displayed secondary effects following peripheral or systemic inflammation, or if the combination of them both led to the neuroinflammation [29].

The pro-inflammatory effects of PM may mediate the indirect neurotoxicity. Activated microglia release neurotoxic substances including cytokines, ROS, complements and other enzymes. TNF-α released from PM-exposed microglia inhibited the neurite outgrowth in a primary culture of cortical neurons [90]. Chronic exposure to nanosized PM showed an increased expression of complement C5/C5 α protein and CD88, the complement component C5α receptor 1, and activated microglia in the corpus callosum in mice [91]. Conditioned medium of PM2.5-treated microglial culture significantly induced neurotoxicity because microglia released glutaminase to generate glutamate leading to excitotoxicity [66]. In a co-culture of neurons and oligomer Aβ stimulated-microglia, PM2.5 aggravated oligomer Aβ-induced neuronal injury by increasing IL-1β through the activation of the NOD-like receptor family pyrin domain-containing 3 inflammasome [92]. This study used a cell model that mimicked the interaction of neurons and microglia in the AD brain to demonstrate that PM2.5 may promote the progression of AD pathology [92].

### Damage to the BBB and NVU

The BBB is created by the endothelial cells that form the walls of the capillaries. Tight junctions are formed at the margins of endothelial cells which seal the aqueous paracellular diffusional pathway between these cells. The integrated tight junctions form a physical barrier to the brain that helps shield the CNS from neurotoxic substances circulating in the blood. The disruption of tight junctions contributes to the increased permeability of the BBB and subsequently results in an imbalance in the CNS homeostasis and worsened disease progression [93]. A key component of the effects of air pollution in the brain is the breakdown of epithelial and endothelial barriers, especially the BBB, because airborne pollutants induce neuroinflammation leading to the robust production of antibodies against the integral proteins in the tight junctions of the BBB. Children living in MCMA showed significantly higher levels of antibodies against tight junctions and neurons in the serum. An increased amount of immunological attacks against the BBB in these children suggests a disturbed microenvironment in the brain, which may attribute to oxidative stress, inflammation, innate and adaptive immune responses, and even the early AD pathology [94]. A study showed that the inhalation of traffic-generated air pollutant increased the BBB’s permeability and decreased the composition of tight junction proteins in the cerebral vessels in *Apo E*^*−/−*^mice [95]. A microfluidic-based 3D culture of endothelial cells and astrocytes mimicked the BBB in vitro. PM2.5 collected from indoors passed through it and triggered oxidative stress and neuroinflammation [30]. Various kinds of artificial nanoparticles, such as nanosized PM, increased the BBB’s permeability in a size-dependent manner [96, 97].

NVU are composed of vascular cells which include endothelial cells, pericytes, and vascular smooth muscle cells, glial cells including astrocytes, microglia and oliogodendroglia, and neurons. The microvessels provide oxygen and nutrient supplies to the neurons, and remove carbon dioxide and other potentially toxic metabolites from the brain’s interstitial fluid. They also control the permeability of the BBB, cerebral blood flow, and maintain the chemical composition of the neuronal milieu, which is required for the proper functioning of neuronal circuits. Neurodegenerative disorders such as AD are often associated with microvascular dysfunction and/or degeneration in the brain, neurovascular disintegration, and/or dysregulation of vascular factors [98]. A study which collected the cerebrospinal fluid from 133 volunteers in the US revealed that short-term [1 year] and long-term (7 years) exposure to high level of PM2.5 was associated with vascular damage in cognitively normal individuals [99]. Such correlation was not seen in patients with mild cognitive impairment or AD, suggesting endothelial damage induced by PM2.5 may be masked by the existing vascular injury caused by the diseases [99]. Endothelin (ET) can induce fast and prolonged constriction of the blood vessels while nitric oxide dilates them. Rats that inhaled a mixture of PM and ozone, which represents a common combination in airborne pollution, for 4 h showed a significant increase in the expression of ET-1 mRNA in the pituitary gland 24 h afterwards, and an initial decrease followed by an increase 24 h later in the mRNA expression for inducible nitric oxide synthase in the cerebral hemisphere, suggesting a dysregulation of vasoregulatory pathways in the brain and pituitary gland [100].

### Subcellular effects of PM on mitochondria and ER

The dysfunction and stress response in mitochondria and ER played essential role in the pathological progression of neurodegeneration by inducing oxidative stress, energy deprivation and misfolded protein deposition, which may subsequently result in neuronal dysfunction and even death. Ultrastructural changes in mitochondria and ER were found in the brains of young citizens of Mexico City, a highly polluted megacity. Such pathological changes were associated with the deposition of combustion-derived nanoparticles in neurons, glia, choroid plexus and NVU according to the observation in transmission electron microscopy [101]. These findings indicated that mitochondria and ER may be susceptible to the insults of PM and its components, and the ultrastructural changes in mitochondria and ER may mediate the neurotoxicity of PM. More specifically, mitochondria which showed abnormal morphology, nanoparticles deposition and disrupted mitochondria-ER contacts were observed in neurons, both soma and dendrites, from the frontal cortex of individuals in Mexico City [101]. Mitochondrial dysfunction played essential role in the neurotoxicity and the pathological progression of neurodegeneration. Several experimental studies confirmed the detrimental effects of PM on mitochondria in neuronal culture and animal expeirments. Human SH-SY5Y cells that were exposed to PM showed swelling morphology, mitochondrial permeability transition pore opening, elimination in mitochondrial potential, reduce in ATP production, decline in mtDNA copy number, and increased dynamics signaling in mitochondria [102]. These findings indicated that PM exposure lead to mitochondrial damage which may subsequently result in energy deprivation, oxidative stress, and eventually mitochondria-dependent apoptosis in neurons. Consistently, ultrastructural changes of mitochondria were also observed in the PM2.5-exposed rats’ brains, which were accompanied with myelin sheaths and cognitive dysfunction [103].

Mitochondrial DNA (mtDNA) may also be affected by exposure to PM. MtDNA is a small independent circular genome, which is the primary source of endogenous oxidative stress and lacks an efficient DNA repair system. Hence, mtDNA may be sensitive to PM-induced toxicity and its damage may contribute to mitochondrial dysfunction. MtDNA damage indicated by increased DNA methylation was associated with the exposure to metal-rich PM in buffy coats of human blood samples [104] and in cell lines such as BEAS-2B cells [105]. Haplogroups in mtDNA mark individual differences in oxidative potential and are possible determinants of neurodegeneration. It was found that the susceptibility of cognitive impairment induced by carbon black in humans is higher in carriers of phylogenetically-related mtDNA haplogroups in Cluster 4, indicating that mitochondria is not only affected by PM but may also regulate the outcome of PM exposure [106].

ER has a vital role in the folding and maturation of newly synthesized secretory and transmembrane proteins [107]. The stress response caused by PM, including neuroinflammation, oxidative stress, and mitochondrial dysfunction, may result in the ER stress in the brain. The ER was abnormally dilated and deposited with external nanoparticles in the neurons of the brains of Mexico city, the heavily polluted urban area, suggesting airborne particulate pollutant may disturb the homeostasis of ER and subsequently induce ER stress [108]. In the striatum and hippocampus of rat brains exposed to coarse PM for 8 weeks, there was a significant increase of X box binding protein-1, indicating the activation of the unfolded protein response [76]. These studies in human and animals suggested that PM exposure may revoke ER stress in the brain. Subsequently, ER stress may mediate pathological changes seen in neurodegeneration, such as neuroinflammation, deposition of misfolded proteins, and neuronal death. The effects of PM on ER stress may contribute to the abnormal deposition of Aβ and α-synuclein in the brains of children from MCMA, leading to the susceptibility of late-life AD and onset of PD [15]. By using in situ Fourier transform infrared microspectroscopy to study the conformation of the proteins in the brain, proteins with β-sheet structure, a form of misfolded protein that are commonly seen in neurodegenerative diseases such as AD, accumulate in the perivascular space in the brains of the offsprings that were maternally exposed to carbon black nanoparticles [109]. These misfolded proteins may be in the process of clearance from the brain [109], which may activate the microglia and astrocytes. Further analysis showed that perivascular macrophages and astrocytes were activated and showed ER stress in the offsprings’ brains when they were maternally exposed to carbon black nanoparticle [110]. These findings indicated that ER stress may mediate the vascular dysfunction, misfolded protein deposition, and neuroinflammation in the brains of offsprings that were maternally exposure to carbon black. ER stress may also lead to the apoptosis of neurons. In the cell culture of SH-SY5Y cells, PM2.5 exposure increased cellular calcium level and apoptosis mediated by upregulation of CHOP/Caspase12/DR5/Caspase8 signaling [111]. These results suggest that exposure to particulate airborne pollutant may induce ER stress, which may subsequently mediate neurotoxicity, neuroinflammation, and misfolded protein deposition.

DNA damage is a crucial factor in aging and likely to contribute to the neuronal dysfunction and progression of age-associated diseases, such as AD. After PM exposure, DNA damage may be induced by both endogenous and exogenous sources, mostly from intermediates of oxygen reduction that either attacks the bases or the deoxyribosyl backbone of DNA. It was found that the apurinic/apyrimidinic sites in genomic DNA, a marker for base excision repair, in the olfactory bulb and hippocampus were significantly increased in the brains of dogs which were naturally exposed to air pollution in MMC [84].

## Summary and conclusion

**Fig. 1:**
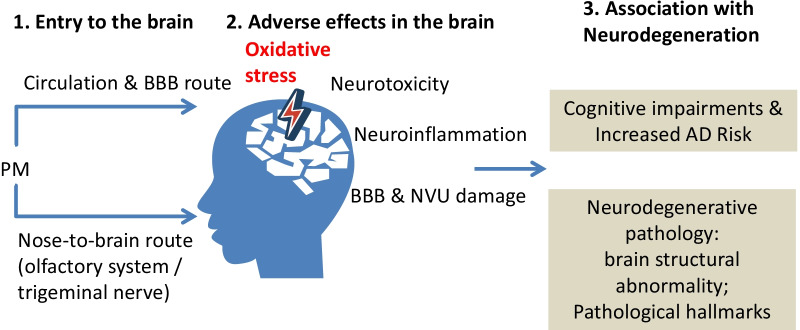
Schematic summary: Airborne PM may enter the brain, inducing neurotoxic effects in the brain and is associated with increased risk of neurodegeneration. This review summarized the studies that linked PM exposure to increased risk of neurodegeneration. Firstly, the airborne PM can enter the brain via either the olfactory route which involves the olfactory system or the trigeminal nerve, or the passing through BBB after enter the circulation. Then the deposited PM can induce neurotoxicity, oxidative stress, neuroinflammation, and BBB and NVU damage. These adverse effects in the brain may result in neurodegenerative pathology, such as brain structural abnormality and pathological hallmarks for neurodegenerative diseases. Epidemiological studies has associated PM exposure with cognitive impairment and increased risk of developing neurodegenerative diseases such as AD. *AD* Alzheimer’s disease, *BBB* blood–brain barrier, *NVU* neurovascular units, *PM* Particulate matter

As summarized in this review, despite the differences among various studies in the source and chemical composition of PM, existing findings from epidemiological and experimental studies consistently suggested a strongly link between chronic exposure to PM, especially PM2.5 and UFPM, with the onset of dementia and AD, as well as neurodegenerative-like pathology and cognitive deficits. The central role of oxidative stress was highlighted in the neuronal injury caused by PM. Neuroinflammation could further damage the neurons and other cells such as the endothelial cells in the NVU (Fig. 1). Targeting the HMGB1/TLR4/NF-κB pathways or oxidative stress by pharmacological inhibitors or genetic knockdown has demonstrated potential as an effective therapeutic intervention against the pathological changes in the brain after exposure to PM.

There are several questions remain to be answered. Firstly, although experimental studies have demonstrated the existence of PM in the brain, the transmission of PM within the brain, that is whether such transmission requires mediates such as vesicles including exosomes or synaptosomes, or activated microglia that phagocytoses with PM, remains obscure. In vivo imaging with a high resolution may help visualize the dynamic transmission and distribution of PM in the brain. Investigating this transmitting process may lead to a new strategy for prevention and therapy against the adverse effect of PM in the brain. Secondly, short-term exposure to PM triggers the stress response in the brain and long-term exposure results in AD-like cognitive impairment and neurological changes. However, the time course of AD-like changes induced by PM exposure is unknown. Thirdly, we could not precisely predict the susceptibility of cohorts that were differentially exposed to PM, nor determine the proper time frame for the intervention. Fourth, to develop therapeutic interventions against the adverse effects of PM exposure in the brain, we must first comprehensively understand the specific molecular mechanisms of PM contributing to AD-like pathology, including the mechanisms of the receptors, signaling pathways, and target organelles. An unbiased study analyzing the whole genome transcriptomics, proteomics, or single-cell/synapse RNA sequencing would further our understanding of the impact of PM on the brain. Lastly, the effects of PM on metabolism should be further studied according to the results in the neurometabolomics analysis. Because this study not only showed the crucial implication of disturbed glutathione metabolism in the pathogenesis of PM-induced neuronal injury but also demonstrated that PM may affect the fatty acid and energy metabolism in the neurons [74]. Injury in the NVU after exposure to PM would also impair energy metabolism in the affected brain regions. Therefore, the disturbed metabolic homeostasis may also play a crucial pathogenic role in the development of PM-induced neuropathology. Restoring these metabolic disturbances may enhance the resistance of neurons against the stress caused by exposure to PM.

Since the problem of PM pollution is unlikely to be solved for decades to come, efforts that may help minimize the health care burden caused by PM are urgently needed.

## Data Availability

Not applicable.

## Abbreviations

AD: Alzheimer’s disease
APP: Beta-site amyloid precursor protein
Aβ: Amyloid-β
BACE: Beta-site amyloid precursor protein cleaving enzyme
BBB: Blood–brain barrier
CA1: Cornuammonis area 1
CNS: Central nervous systems
ER: Endoplasmic reticulum
ET: Endothelin
HMGB1: High-mobility group box 1
HRTEM: High-resolution transmission electron microscopy
MCI: Mild cognitive impairment
MCMA: Mexico City Metropolitan Area
MMC: Metropolitan Mexico City
mtDNA: Mitochondrial DNA
NF- ĸb: Nuclear factor-ĸb
Nrf2: Nuclear factor (erythroid-derived 2)-like 2
NVU: Neurovascular units
PD: Parkinson’s disease
PM: Particulate matter
ROS: Reactive oxygen species
TLR4: Toll-like receptor 4
UFPM: Ultrafine particulate matter
